# Description, molecular identification and pathological lesions of *Huffmanela persica* sp. nov. (Nematoda: Trichosomoididae: Huffmanelinae) from the daggertooth pike conger *Muraenesox cinereus*

**DOI:** 10.1186/s13071-023-05772-7

**Published:** 2023-06-05

**Authors:** Reza Ghanei-Motlagh, Mark D. Fast, David Groman, Gokhlesh Kumar, Hatem Soliman, Mansour El-Matbouli, Mona Saleh

**Affiliations:** 1 Division of Fish Health, University of Veterinary Medicine, 1210 Vienna, Austria; 2 Department of Pathology and Microbiology, Atlantic Veterinary College, University of Prince Edward Island, Charlottetown, PEI Canada; 3 Aquatic Diagnostic Services, Atlantic Veterinary College, University of Prince Edward Island, Charlottetown, PEI Canada; 4 Department of Aquatic Animal Medicine, Faculty of Veterinary Medicine, Assiut University, Assiut, 71515 Egypt

**Keywords:** *Huffmanela*, Marine fish, *Muraenesox cinereus*, Molecular identification, Histopathology

## Abstract

**Background:**

The genus *Huffmanela* Moravec, 1987 (Nematoda, Trichosomoididae, Huffmanelinae), represents a group of nematodes that infect both marine and freshwater fish, and the main gross feature of infection with different species of the genus is the presence of noticeable dark spots or tracks within the parasitized tissues. The purpose of this study was to describe morphologically and morphometrically the eggs of a new marine species of *Huffmanela* (*Huffmanela persica* sp. nov.), which was found in the form of black spots in the ovary and the tunica serosa of the stomach of the daggertooth pike conger (*Muraenesox cinereus*). The new species differs from *Huffmanela hamo*, another species reported from musculature of this host in Japan, in egg metrics, eggshell features and targeted organ. Molecular identification and pathological examination of the lesions caused by the new species are also reported.

**Methods:**

Nematode eggs with varying degrees of development were separated from the infected tissues (ovary and tunica serosa of stomach) and investigated using light and scanning electron microscopy. Different species-specific markers (small subunit ribosomal DNA, 18S; large subunit ribosomal DNA, 28S; internal transcribed spacer, ITS) were used for molecular identification and phylogenetic study of the new species. Infected tissues were fixed in buffered formalin for pathological investigations.

**Results:**

The fully developed eggs of *H. persica* sp. nov. are distinguished from those previously described from this host on the basis of their measurements (size, 54–68 × 31–43 µm; polar plugs, 6.4–9.7 × 8.4–12 µm; shell thickness, 3.5–6.1 µm) and a delicate but ornate uterine layer (UL) covering the entire eggshell including the polar plugs. Histopathological examination revealed a fibro-granulomatous inflammation in the ovary and the serosal layer of the stomach of infected fish. Maximum-likelihood (ML) phylogenetic analysis recovered a sister relationship between the new species of marine origin and *Huffmanela* species previously collected from freshwater hosts.

**Conclusions:**

The present study is the first to report the molecular characterization and phylogenetic position of a teleost-associated marine species of the genus *Huffmanela*. A comprehensive list of nominal and innominate populations of *Huffmanela* is also provided.

**Graphical abstract:**

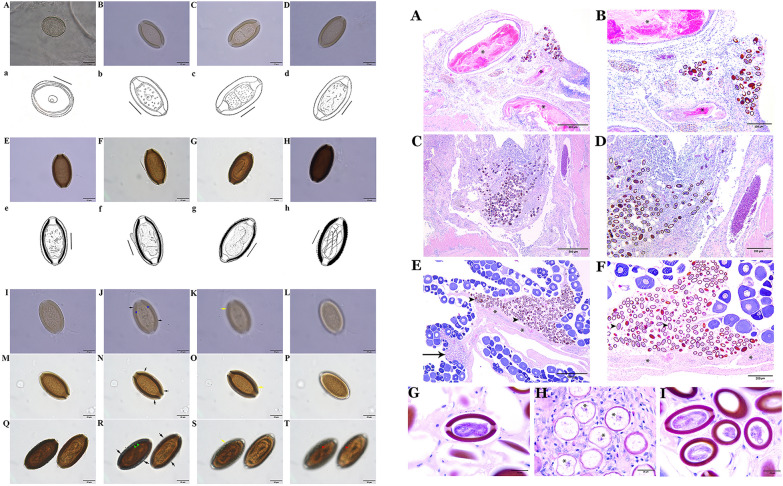

## Background

Huffmanellosis, which is known to occur in a variety of fish species inhabiting marine and freshwater environments, is a parasitic infection caused by members of the genus *Huffmanela* Moravec, 1987 (Nematoda, Trichinelloidea, Trichosomoididae, Huffmanelinae) [1]. To date, 23 nominal species and approximately 12 innominate species of the genus *Huffmanela* have been described or reported, most of which were distinguished based on morphology of their remarkable eggs (eggs generally are regarded as syntypes) without collecting the rarely found adult worms [2]. Many previously recognized congeneric species were collected from marine fishes distributed across global ocean ecosystems. Infections due to freshwater species of *Huffmanela* were, however, only reported in certain fish belonging to Centrarchidae and Poeciliidae in the USA and Tetraodontidae in Brazil [3–6]. The amphipod crustacean *Hyalella* serves as the experimental intermediate host in the life cycle of freshwater populations of *Huffmanela* (*Huffmanela huffmani*, Moravec, 1987) [7, 8]. There is lack of information regarding the life cycle and biology of marine species of *Huffmanela*, but closely related amphipods of marine origin probably act as their intermediate hosts [9]. In general, eggs are ingested by the intermediate host, and then by a fish as the definitive host, where the larval nematodes mature to the adult forms. After mating, the adult females lay numerous eggs within the species-specific tissues, and shortly afterwards the adult nematodes begin to disappear [3, 10]. Eggs continue to develop and appear mostly as grossly visible dark spots in the infected sites [11, 12]. The presence of dark discolored spots or tracks originated from eggs deposited in fish tissue as the result of movements of adult female worm is pathognomonic of *Huffmanela* infection [13]. Extra-intestinal infections with eggs and adult forms of the histozoic parasite *Huffmanela* have been recorded in a diverse range of teleost and elasmobranch fish, mainly in skin, swim bladder, serous membrane, mesentery, muscle, gonads and bone [14, 15].

Generally, huffmanellosis is not a severe problem in wild fish. Nonetheless, infections observed in external organs and musculature (flesh) could negatively impact the marketability of affected fish and result in their rejection by final consumers [16]. In addition, infection diagnosed in the swim bladder was found to reduce the physiological efficiency of this specific organ [5]. Eggs associated with *Huffmanela* species have also been detected in stool samples collected from humans; however, there is no report on the zoonotic potential of *Huffmanela* species from a public health perspective [17–19].

In the present study, we describe a new species of *Huffmanela* (*Huffmanela persica* sp. nov.) from the daggertooth pike conger (*Muraenesox cinereus*) according to morphometric and morphological examinations of the parasite eggs using light and scanning electron microscopy, provide for the first time to our knowledge the molecular sequence data associated with three different genetic markers (18S, 28S and ITS) in a teleost-associated marine species of *Huffmanela* and report the pathological lesions caused by naturally occurring infection with the nematode in the host tissues.

## Methods

### Sample collection

During January 2021, a total of 20 freshly caught fish (*M. cinereus*) with average length 86.65 ± 7.74 cm (mean ± SD) were purchased from local fishermen off the coast of Zir Ahak, Bushehr, Iran (28°17' N, 51°13' E). Gross and stereomicroscopic examinations of fish and their organs were respectively performed to detect any parasitic infection. While no adult specimen of *H. persica* sp. nov. was recovered from the internal organs, the ovary and the serous coat (tunica serosa) of the stomach were found to be infected by discernible dark egg clusters deposited by the female nematode. Infected tissues containing eggs were collected and divided into two portions. The first portion was stored in 10% neutral buffered formalin for morphological and pathological examinations. The second portion was preserved in 70% ethanol for molecular and SEM analyses [15]. Simultaneously, eggs were scraped from unfixed infected tissues with a scalpel, wet mounted and cover slipped on glass slides, and subjected to gentle pressure before being fixed in formalin for measurement of the size of nematode larvae [10, 20].

### Morphological and morphometric analyses

**Fig. 1:**
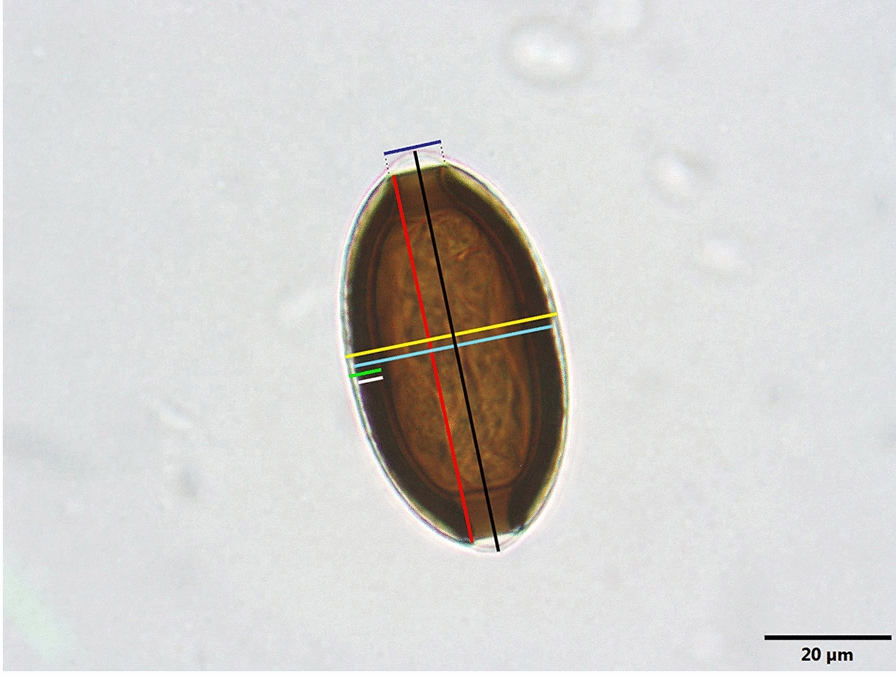
Representation of an advanced egg of *Huffmanela persica* sp. nov. with measurements considered in this study. Total length with protruding polar plug and UL (black line); total length without protruding polar plug and UL (red line); total width with UL (yellow line); total width without UL (light blue line); shell thickness with UL (green line); shell thickness without UL (white line); polar plug width (dark blue line)

To investigate the morphology and morphometry of the eggs, egg clusters within the infected tissues were separated, cleared in glycerol and mounted in glycerin jelly (for temporary mounting) or Canada balsam (for permanent mounting). All measurements were performed using cellSens imaging software integrated with a digital camera (Olympus SC50 CMOS) installed on a compound microscope (Olympus BX-53). Egg measurements and morphological characteristics considered in the present study (see Fig. 1) were according to those previously described [15, 21–24]. To describe the architecture of the eggs of *H. persica* sp. nov., the novel anatomical and terminological framework proposed by Bond and Huffman, 2023, was adopted [25]. Measurements are given in micrometers as range (minimum–maximum) followed by mean ± standard deviation (SD) in parentheses. Line drawings were made with the aid of a drawing tube.

### Scanning electron microscopy

Nematode eggs fixed in 70% ethanol (stored at 4 °C) were carefully separated, transferred into 70% acetone overnight and dehydrated in an ascending series of acetone ranging from 70 to 90% at 10-min intervals followed by anhydrous acetone three times for 30 min [15]. The samples were subsequently treated with a mixture (1:1 v/v) of anhydrous acetone and hexamethyldisilazane (HMDS, Sigma-Aldrich, Germany) and immersed in HMDS (as the final desiccation step) three times for 60 min each. After several hours of air drying, they were mounted on metal stubs using double-sided adhesive tape, sputter coated with a thin layer (4 nm) of gold in a sample coater (Balzers SCD 050) and examined with a tabletop scanning electron microscope (Hitachi TM-1000, operated at accelerating voltage of 15 kV) equipped with a high-sensitive semiconductor BSE detector.

### Preparation of histopathological sections

Infected tissues fixed in formalin were rinsed in water, dehydrated in ascending grades of ethanol, cleared in xylene and impregnated in paraffin. The specimens embedded in paraffin were micro-sectioned at 4 μm thickness using a microtome (Microm HM 360 microtome, MICROM Laborgeräte GmbH, Walldorf, Germany), double stained with hematoxylin and eosin (H&E) and mounted on glass slides [26]. The histological sections were examined using light microscopy and the images were taken with optical magnifications 5×, 10× and 100× (Leica DM 2500).

### DNA barcoding

Egg aggregations were removed from the infected tissues preserved in 70% ethanol, transferred into 2-ml Eppendorf tubes, rinsed in nuclease-free water and collected by centrifugation at 9660×*g* for 1 min. Genomic DNA from the nematode eggs was extracted using the DNeasy Blood and Tissue kit following the manufacturer’s instructions (Qiagen GmbH, Hilden, Germany). Briefly, 180 μl of the lysis buffer ATL and 40 μl of proteinase K (20 mg/ml) were added to each tube containing collected eggs, and the tubes were vortexed for 15 s and incubated overnight on an Eppendorf Thermomixer R (Hamburg, Germany) at 56 °C. Thereafter, 200 μl of the lysis buffer AL was added to each tube followed by incubation at 56 °C for 10 min. The unlysed chitinous material from the eggs was precipitated by centrifugation at 9660×*g* for 30 s, and the supernatant containing the DNA was transferred to a new 2-ml Eppendorf tube. A total of 200 μl of pure ethanol was added, and the mixture was pipetted into a DNeasy Mini spin column placed in a 2-ml collection tube. After two washing steps using the wash buffers AW1 and AW2, the genomic DNA was eluted in a 1.5-ml tube by adding 35 μl of the buffer AE. Total DNA was also extracted from uninfected host tissue and included in the polymerase chain reaction (PCR) reactions as a negative control. Concentration and purity of the extracted DNA were measured using a NanoDrop 2000 (Thermo Scientific, USA). The concentration of purified DNA from the eggs was found to be 1050 ng/µl and subsequently adjusted at 100 ng/µl before being used for PCR reactions. The 260/280 and 260/230 absorbance ratios were 2.11 and 2.18, respectively. Three different markers were used for molecular identification of the parasite. The 18S rDNA gene was partially amplified by PCR using the primers Nem_18S_F and Nem_18S_R as described previously [27]. PCR amplification of the 28S rDNA was performed using the primer sets D2A and D3B and 28SF and 28SR [28, 29]. The entire ITS region comprising the ITS1, 5.8S gene and the ITS2 was amplified by PCR using the primers NC5 and NC2 [30]. The primer sequences used are listed in Table 1. PCR reactions were performed in 50 µl reaction mixture composed of 25 µl DreamTaq Green PCR master mix (Thermo Scientific), 2 µl 10 pmol/ul forward and reverse primers, 1 µl 25 mM MgCl_2_ (Thermo Scientific), 15 µl nuclease-free water and 5 µl 100 ng/µl DNA. PCR cycling conditions used in this study were those optimized in previous studies [27–30]. PCR products were verified on 1% agarose gel, excised from the gel and purified using a MinElute Gel Extraction Kit as per the manufacturer’s protocol (Qiagen GmbH, Hilden, Germany). As purified PCR product amplified from the entire ITS region (ITS1, 5.8S and ITS2) was not successfully sequenced, it was cloned into the pCR™4-TOPO^®^ TA vector using a commercially available kit (TOPO^®^ TA Cloning^®^ Kit, Invitrogen, USA). The resultant construct was transformed into competent cells (Invitrogen™ One Shot™ TOP10 Chemically Competent *E. coli*), and transformants were plated on Luria–Bertani (LB) agar supplemented with 50 µg/ml kanamycin. Positive clones were then analyzed by colony PCR screening, and plasmid DNA was isolated using the QIAprep Spin Miniprep Kit according to the protocol included in the manual. Purified DNA and plasmid samples were sequenced in both orientations using the same primers used in PCR reactions. Barcode sequencing was carried out using an automated sequencer (ABI 3730 XL) at LGC Biosearch™ Technologies (LGC Genomics GmbH, Berlin, Germany). To extract consensus sequences related to each gene, the obtained sequence files were aligned using the software MEGA X, checked by the program Chromas version 2.6.6 and assembled manually [31].

**Table 1:** List of primers used in this study

Molecular marker	Primer set	Sequence (5′-3′)	References
18S rDNA	Nem_18S_F	CGCGAATRGCTCATTACAACAGC	[27]
	Nem_18S_R	GGGCGGTATCTGATCGCC	
28S rDNA	D2A	ACAAGTACCGTGAGGGAAAGT	[28, 29]
	D3B	TGCGAAGGAACCAGCTACTA	
	28SF	AGCGGAGGAAAAGAAACTAA	
	28SR	ATCCGTGTTTCAAGACGGG	
ITS region	NC5	GTAGGTGAACCTGCGGAAGGATCATT	[30]
	NC2	TTAGTTTCTTTTCCTCCGCT	

### Phylogenetic analysis

Standard nucleotide BLAST (blastn) was used to compare the target sequences (18S, 28S and ITS) with sequence data previously registered in GenBank repository. All available sequences of different species of genera belonging to the family Trichosomoididae Hall, 1916, and sequence data representing species of important genera within the other known families of the superfamily Trichinelloidea Ward, 1907 (Capillariidae Railliet, 1915; Trichinellidae Ward, 1907; Trichuridae Ransom, 1911; and Cystoopsidae Skryabin, 1923), were retrieved from GenBank and included in phylogenetic analysis (Table 2) [5, 32, 33]. Likewise, only sequences with approximately similar length of those newly generated in this study were selected. A phylogenetic tree was constructed based on the 18S rDNA dataset obtained (as 28S rDNA and ITS sequences related to *Huffmanela* and its closely related taxa have not been yet deposited in the GenBank sequence database) by maximum likelihood (ML) method using MEGA X software [34]. Multiple alignments were performed using ClustalW implemented in the same software with default parameters. Alignments were then trimmed (at both ends) to the shortest sequence length and best-fit nucleotide substitution model was selected [35, 36]. ML analysis was carried out following the substitution model K2 + G (Kimura 2-parameter with gamma distribution) with 1000 bootstrap replications.

**Table 2:** List of GenBank accession numbers for sequence data used in phylogenetic analyses in this study

Species	Accession number (18S rDNA)	References
*Capillaria anatis*	LC052335	[77]
*Capillaria bursata*	LC425006	[78]
*Capillaria madseni*	LC052348	[77]
*Capillaria pudendotecta*	LC052343	[77]
*Capillaria spinulosa*	LC424999	[78]
*Capillaria suis*	LC052376	[77]
*Capillaria tenuissima*	EU004822	[79]
*Huffmanela* cf.* huffmani*	ON838247	[5]
*Huffmanela* cf.* huffmani*	ON838248	[5]
*Huffmanela huffmani*	ON838249	[5]
*Huffmanela huffmani*	ON838251	[5]
*Huffmanela markgracei*	ON838250	[5]
*Huffmanela persica*	OQ418445	This study
*Huffmanela* sp.	ON838246	[5]
*Pseudocapillaria tomentosa*	KU987805	[80]
*Trichinella britovi*	AY851257	[81]
*Trichinella murrelli*	AY851259	[81]
*Trichinella nativa*	AY851256	[81]
*Trichinella nelsoni*	AY851261	[81]
*Trichinella papuae*	AY851263	[81]
*Trichinella patagoniensis*	MF628272	[82]
*Trichinella spiralis*	U60231	[83]
*Trichinella zimbabwensis*	AY851264	[81]
*Trichosomoides crassicauda*	LC425007	Unpublished
*Trichuris arvicolae*	HF586908	[84]
*Trichuris discolor*	HF586910	[84]
*Trichuris muris*	AF036637	[85]
*Trichuris ovis*	HF586911	[84]
*Trichuris skrjabini*	HF586912	[84]
*Trichuris suis*	HF586905	[84]
*Trichuris trichiura*	LC596914	Unpublished
*Trichuris vulpis*	HF586909	[84]

## Results

### *Huffmanela persica* sp. nov.

#### Taxonomic summary

*Type definitive host*: Daggertooth pike conger, *Muraenesox cinereus* (Forsskål), Anguilliformes, Muraenesocidae, date of collection, February 2021.

*Type locality*: Persian Gulf off the coast of Zir Ahak, Bushehr, Iran (28°17' N, 51°13' E).

**Fig. 2:**
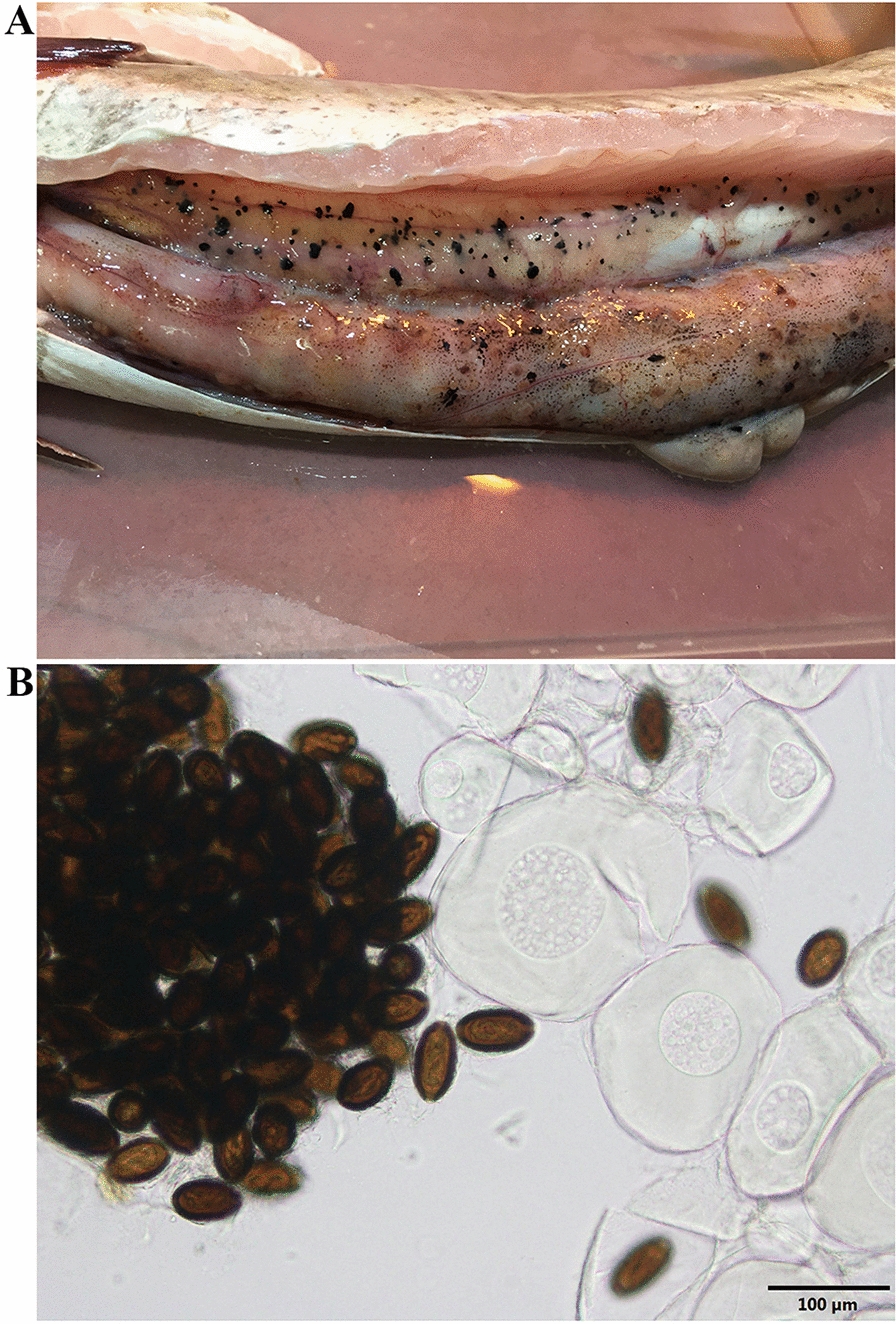
Macroscopic and microscopic appearance of fully developed eggs of *Huffmanela persica* sp. nov. **A** Grossly visible lesions of eggs previously deposited by adult forms of *H. persica* sp. nov. in the form of dark spots of various size within the infected tissues (ovary and serosa of stomach) of *Muraenesox cinereus*. **B** Wet mount prepared from infected ovary illustrating variously oriented advanced eggs of *H. persica* sp. nov. within the egg clusters

*Site of infection*: Ovary, tunica serosa of stomach (Fig. 2A). Conspicuous masses of eggs of *H. persica* sp. nov. were observed in the ovaries of all 13 fish infected. The egg aggregations were simultaneously found in the serosae of stomach of 3 out of 13 fish infected by the nematode. All fish examined were also infected by larval (viscera) and adult (stomach and intestine) forms of anisakids and raphidascaridids. Adult forms (male and female) of *H. persica* sp. nov. were not observed.

*Prevalence*: 65% (13 infected out of 20).

*Etymology*: The specific name *persica* (Persian) refers to the country of its occurrence (Iran).

*Molecular characterization*: Nucleotide sequences of the 18S rDNA (OQ418445), 28S rDNA (OQ428648) and ITS rDNA (OQ428649) of the new species have been deposited in GenBank.

*Deposited specimens*: Syntype eggs collected from the host (*M. cinereus*) were deposited in the Helminthological Collection, Institute of Parasitology, Biology Centre of the Czech Academy of Sciences, České Budějovice, Czech Republic (catalog no. N-1277).

## *ZooBank registration*:

To comply with the regulations set out in article 8.5 of the amended 2012 version of the International Code of Zoological Nomenclature [37], details of the new species have been submitted to ZooBank. The Life Science Identifier (LSID) of the article is urn:lsid:zoobank.org:pub:84ABB846-5AF2-4008-97F6-573A68DFD1BB. The LSID for the new species name *Huffmanela persica* is urn:lsid:zoobank.org:act: urn:lsid:zoobank.org:act:E12D7B01-E298-4BEE-AD69-8C075F84B33E.

### Description of eggs

Grossly visible eggs of *H. persica* sp. nov. were mostly observed in the form of black aggregates with different dimensions which were randomly distributed in the infected tissues (Fig. 2B). Scalpel scrapings obtained from fresh and fixed egg clusters showed thousands of variously oriented eggs with untinted, brown or black eggshells depending on the stage of development of each egg. Four stages of egg development (from less developed to fully developed eggs) are reported herein based upon morphology and morphometry of eggs as well as embryonic stages of development observed in eggs. These include stage I (meiotic stage), stage II (early mitotic embryonated stage), stage III (late embryonated stage) and stage IV (vermiform larvated stage).

#### Stage I eggs

**Fig. 3:**
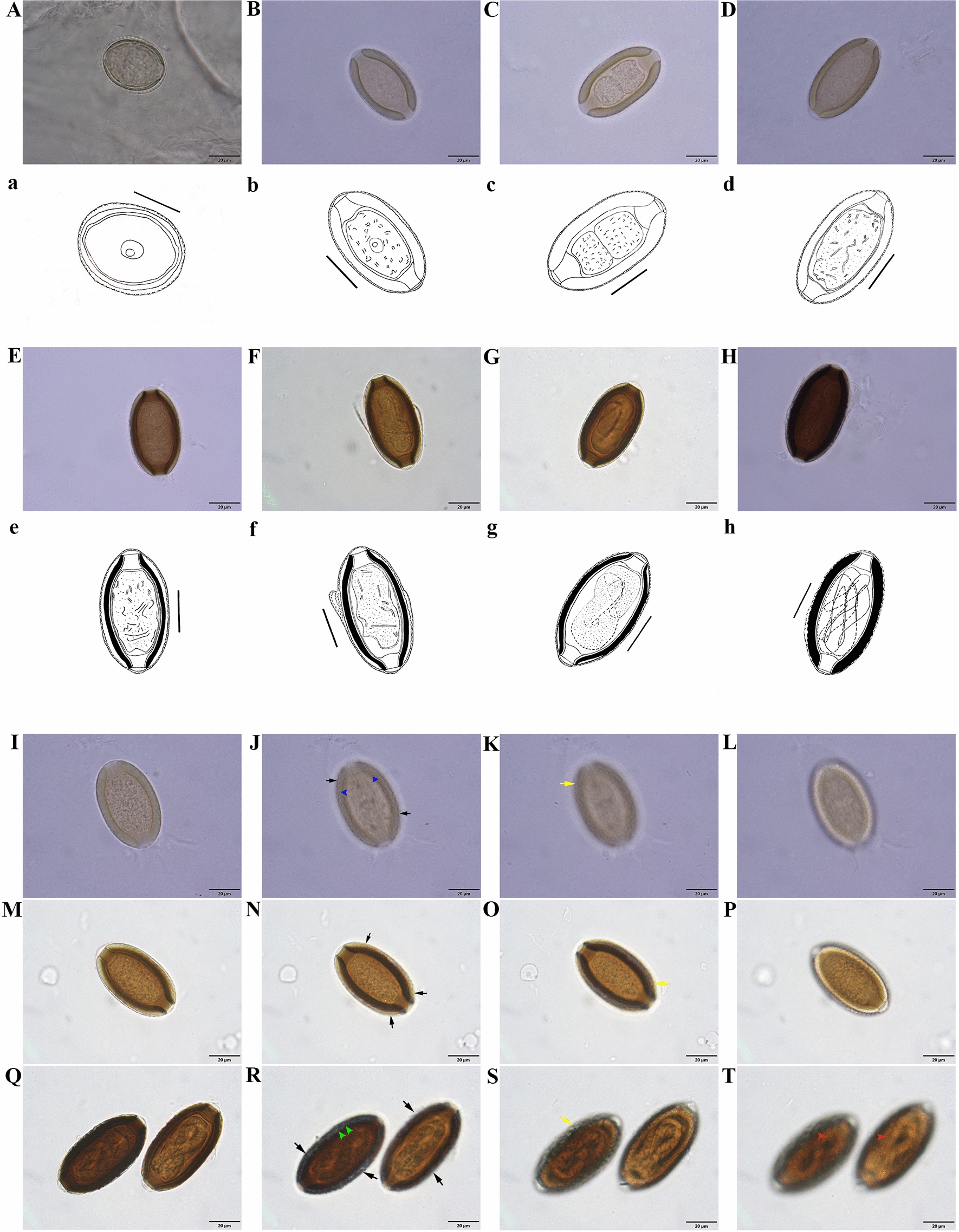
General morphology and surface ornamentation pattern of eggs of *Huffmanela persica* sp. nov. in various stages of development. **A**–**H** Photomicrographs and **a**–**h** corresponding line drawings of individual eggs at different stages of development (scale bars: 20 µm). **A**, **a** Eggs in stage I at very early stage, probably meiosis I, with a spherical nucleus in granular cytoplasm and incompletely developed chitinous layer and polar plugs (note early appearance of superficial projections of UL already apparent). **B**, **b** Eggs in stage II at later stage of development (probably meiosis II); chitin deposition appears to be complete. **C**, **c** Two-celled mitotic stage of early embryonic development (embryonated). **D**, **d** Later multicellular stage of embryonic development; chitinous layer still uniformly translucent with no apparent division into outer and inner chitinous layers. **E**, **e** Eggs in stage III with bean-like embryo and chitinous layer appearing two-layered under bright-field (light) microscopy with darker inner layer. **F**, **f** Tadpole-like embryos with UL appearing to have been partially dislodged from chitinous layer. **G**, **g** Eggs in stage IV with darker-brown shell; embryo now vermiform (larvated) and in-folded three times (pretzel stage). **H**, **h** Later stage IV egg with chitinous layer very dark brown; larva nearing final development and folded 5–6 times. **I**–**T** Photomicrographs of less developed (**I**–**L**), moderately developed (**M**–**P**) and fully developed (**Q**–**T**) eggs, where the first set of images (**I**, **M**, **Q**) represents overview of these variously advanced eggs, and the second (**J**, **N**, **R**), third (**K**, **O**, **S**) and fourth (**L**, **P**, **T**) series of images focus on the pattern of their surface ornamentation by adjusting the focal plane. Black and yellow arrows represent illusions of superficial ridges (well demonstrated in less developed eggs; occasionally appearing as interconnecting ridges, blue arrowhead) and sculptures on the egg surface, respectively. Green arrowheads exhibit irregular protuberances on the eggshell surface. Red arrowheads indicate an illusory spinous appearance in fully developed eggs

**Fig. 4:**
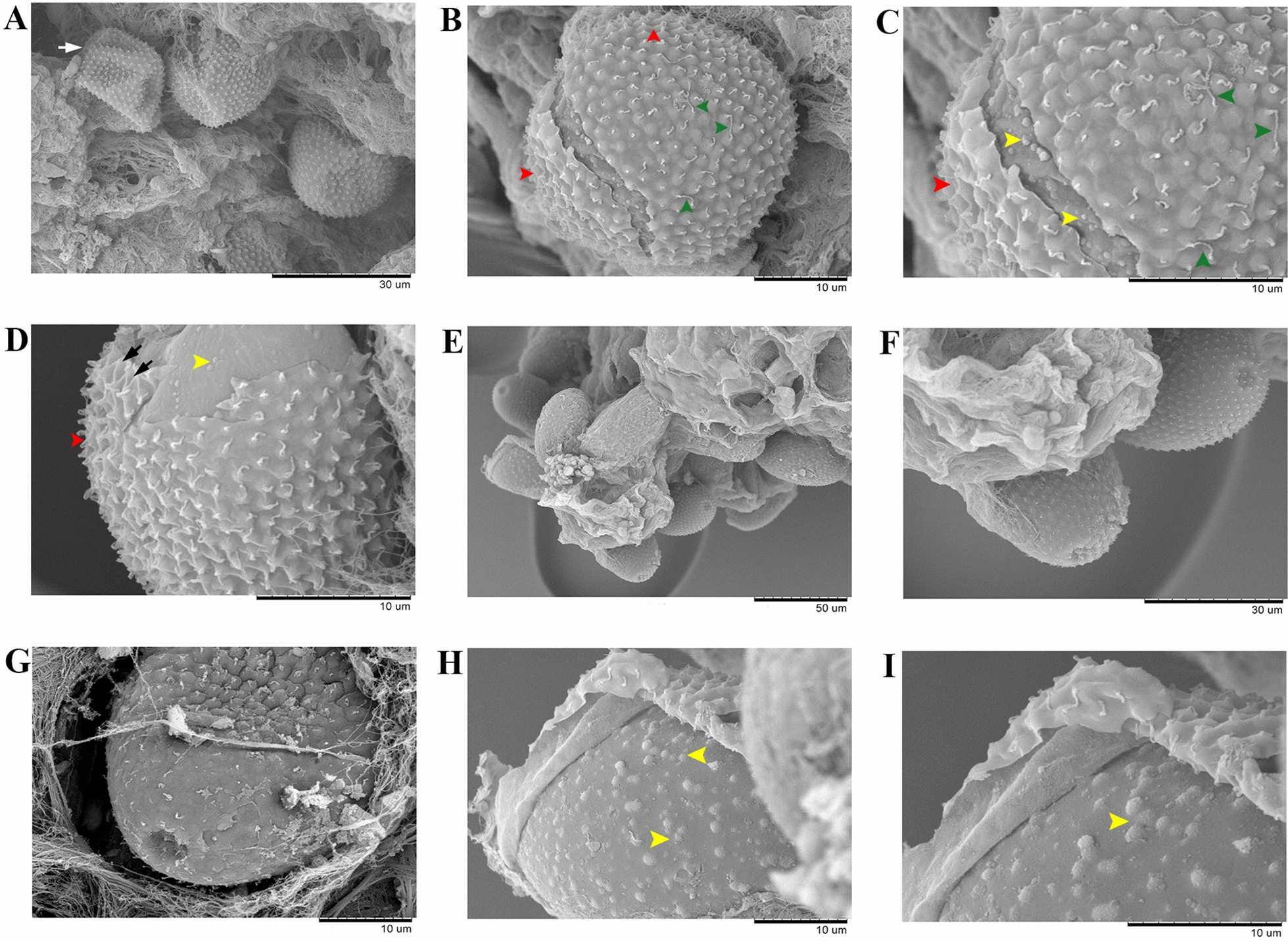
Scanning electron micrographs of poorly developed (**A**–**D**) and fully developed (**E**–**I**) eggs of *Huffmanela persica* sp. nov. (separated from infected ovary). Less developed eggs spherical shaped (white arrow shows a shrunken and wrinkled egg) and with no evidently developed polar plugs. Fully developed eggs oblong and containing two plugs at poles. Eggs completely surrounded by a UL bearing uniformly based mammiform mounds adorned with tendril-like vermiform appendage emerging from the apex, occasionally adjoined to that of a neighboring mound (green arrowheads). Note the illusory appearance of serrated-like ridges (occasionally in the form of illusory interconnecting ridges, black arrows) in side views which is caused by overlapping of the mound bases (well observed in less developed eggs, red arrowheads). Outer surface of eggshell with irregular protuberances (yellow arrowheads)

**Fig. 5:**
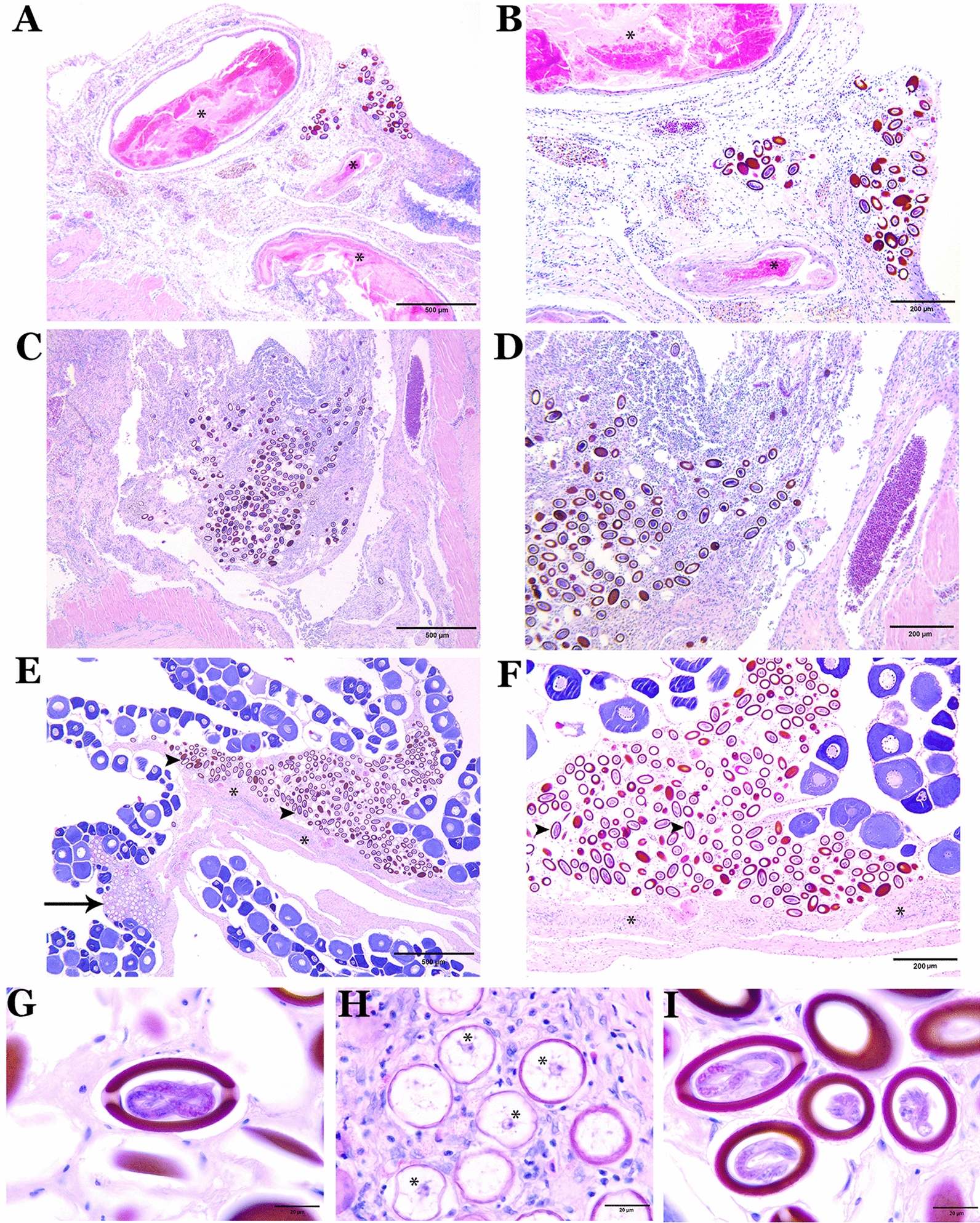
Photomicrographs from histological sections of infected tissues of a daggertooth pike conger eel. **A**, **B** Histological sections of the stomach infected by eggs of *Huffmanela persica* sp. nov. at various stages of development as well as degenerated encysted metazoans (⁎). **C**, **D** Sections of the tunica serosa of the stomach parasitized with completely developed eggs. **E** Sections of the ovarian lamellae infected by both clusters of immature (➔) and developing (➤) eggs, representing immature and previtellogenic oocytes embedded within a loose fibro-granulomatous infiltration containing histiocytes and eosinophilic granular leukocytes (⁎). **F** Magnified view of histological section of infected ovary showing a fibro-granulomatous infiltrate surrounding clusters of eggs at different stages of development (mainly highly developed eggs, ➤). **G** High magnification (100×) view of a fully developed egg of *H. persica* (cross-sectional view) showing larva in-folded within the eggshell and a protruding polar plug at either end. **H** Less developed eggs with a nearly central nucleus (⁎) and thin eggshell layer and **I** fully developed eggs containing twisted larvae of *H. persica* cut on varying planes of section

Stage I eggs (Figs. 3A, a; 4A–D; 5H) were evidently recently laid eggs in probably meiosis I with a distinctly spherical nucleus mainly located centrally in the granular cytoplasm and no developed or two poorly developed plugs at poles. Eggs in stage I spherical shaped (occasionally shrunken and wrinkled), with light amber rigid eggshell walls and no discernable layering. Egg morphometrics (*n* = 15) recorded in stage I was as follows: total length (with uterine layer, UL) 34.14–44.32 (38.12 ± 2.97); total length (without UL) 30.51–39.51 (34.06 ± 2.76); total width (with UL) 32.65–41.25 (35.50 ± 2.06); total width (without UL) 30.45–37.20 (32.60 ± 1.72); shell thickness (with UL) 3.27–6.07 (4.74 ± 0.81); shell thickness (without UL) 1.54–2.99 (2.47 ± 0.44); nucleus length 8.2–10.71 (9.24 ± 0.87); nucleus width 7.57–10.08 (8.53 ± 0.74).

#### Stage II eggs

Stage II represents various stages of early embryonic development within the eggshell from single-cell (probably meiosis II) stage to multiple cell mitotic stages (Fig. 3B, b; C, c; D, d). Eggs in the single-cell stage (Fig. 3B, b) were distinguished by having a spherical nucleus present in the granular cytoplasm and two newly developed polar plugs. Eggs in the two-cell stage (Fig. 3C, c) had a symmetric pattern as the result of the first cell division. Eggs containing multi-cell stages (Fig. 3D, d) showed further development of embryo, where the inner space of the rigid eggshell wall was partly or completely occupied by a multicellular mass. Eggs in stage II were elliptical or oblong, light yellow to light brown, with polar plugs (apparently two-layered) slightly or markedly protruding and no distinct shell layers. Egg morphometrics (*n* = 40) obtained in stage II: total length (inclusive of protruding polar plugs and UL) 46.79–65.84 (58.26 ± 4.83); total length (exclusive of protruding part of polar plugs and UL) 43.24–61.85 (54.08 ± 4.85); total width (with UL) 31.51–40.80 (35.43 ± 2.23); total width (without UL) 30.35–39.52 (33.57 ± 2.35); shell thickness (with UL) 3.43–8.44 (5.20 ± 1.11); shell thickness (without UL) 2.70–7.57 (4.28 ± 1.14); polar plug width 7.52–15.76 (9.78 ± 1.65).

#### Stage III eggs

Eggs in stage III (Fig. 3E, e; F, f) demonstrating higher development of embryo were characterized by possessing spatially differentiated forms (bean-like or tadpole-like embryos; Fig. 3E, e; F, f), two easily distinguishable apparent shell layers and mild to moderate degrees of embryonic bending within the eggshell. Eggs in stage III elliptical or oblong in shape, brown in color, containing two protruding polar plugs, with bilayer shell inclusive of a thin and light (hyaline) inner layer alongside with a thick and dark outer layer (this layering is an optical illusion under light microscopy caused by the birefringent properties of chitin; see Bond and Huffman, 2023) [25]. Morphometric measurements of eggs (*n* = 27) in stage III are summarized as follows: total length (with protruding polar plugs and UL) 59.39–70.98 (62.44 ± 2.60); total length (without protruding part of polar plugs and UL) 54.75–61.67 (57.39 ± 1.94); total width (with UL) 32.46–44.75 (34.95 ± 3.03); total width (without UL) 30.62–43.30 (32.88 ± 3.05); shell thickness (with UL) 3.81–5.73 (4.90 ± 0.52); shell thickness (without UL) 2.92–5.02 (3.77 ± 0.46); polar plug width 8.18–11.09 (9.39 ± 0.68).

#### Stage IV eggs

Stage IV eggs (Figs. 3G, g; H,  h; 4E–I; 5G, I) were differentiated by having brown to dark-brown color, oblong or elliptical shape, varying degrees of larval formation within egg from threefold stage (pretzel, Fig. 3G, g) to five–sixfold stage (fully developed larva, Fig. 3H, h), dark outer layer much thicker in width and two distinctly protruding polar plugs. Morphometric data obtained from stage IV eggs (*n* = 71) were as follows: total length (with protruding polar plugs and UL) 60.37–75.13 (66.62 ± 3.04); total length (without protruding part of polar plugs and UL) 54.37–67.86 (62.03 ± 2.94); total width (with UL) 32.54–44.63 (36.46 ± 2.65); total width (without UL) 30.96–43.39 (34.41 ± 2.78); shell thickness (with UL) 4.48–7.17 (5.63 ± 0.53); shell thickness (without UL) 3.51–6.11 (4.63 ± 0.55); polar plug width 8.43–12.00 (10.29 ± 0.79); larval width in fully developed eggs 6.09–9.16 (7.91 ± 0.61); larval length (*n* = 17) in fully developed eggs 196.27–231.49 (209.51 ± 10.64).

### Egg surface ornamentations

The rigid eggshell wall (or its integral outermost layer, *Pellicula Ovi*) at all stages adorned with irregular protuberances on the surface (Fig. 4B–D, H, I). Eggs thoroughly surrounded by a thin transparent UL decorated with closely and regularly spaced mammiform mounds (superficial projections, Figs. 3 I–T, 4B–D, F, G) appearing to form pointed serrated-like ridges (Fig. 3J, N, R) (occasionally appearing as interconnecting ridges; Figs. 3J, 4D) on the egg surface (the appearance of ridges is most likely a geometric illusion; see Figs. 1–3, 7, 8 of Žďárská et al. 2001, Fig. 30 of Bond, 2020 and Fig. 24 of Bond and Huffman, 2023) [25], resulting in manifestation of a fine superficial sculpture on the surface of eggs examined with light microscope (Fig. 3K, O, S). UL mammiform mounds predominantly with a tendril-like vermiform appendage extending from the tip, sometimes adjoined to that of a neighboring mound (Fig. 4A–F).

### Pathological lesions

The serosal surface of the stomach in infected fish contained eggs of *H. persica* sp. nov. at various stages of development (Fig. 5A–D) and degenerated encapsulated metazoans (Fig. 5A, B; more likely larval forms of *Anisakis* and less likely adult forms of *Huffmanela* as the females are usually extremely long, are rarely found encapsulated and are not much thicker than the eggs), all of which were enclosed in a fibro-granulomatous infiltrate. Ovigerous lamellae of the ovary were infected with nests of developing eggs from the nematode *H. persica*. The eggs were located within both clusters of immature and previtellogenic oocytes and the connective tissue of the lamellae and encased by a fibro-granulomatous infiltrate containing both histiocytes and eosinophilic granular leukocytes (Fig. 5E, F).

### Gene characterization and phylogenetic analysis

**Fig. 6:**
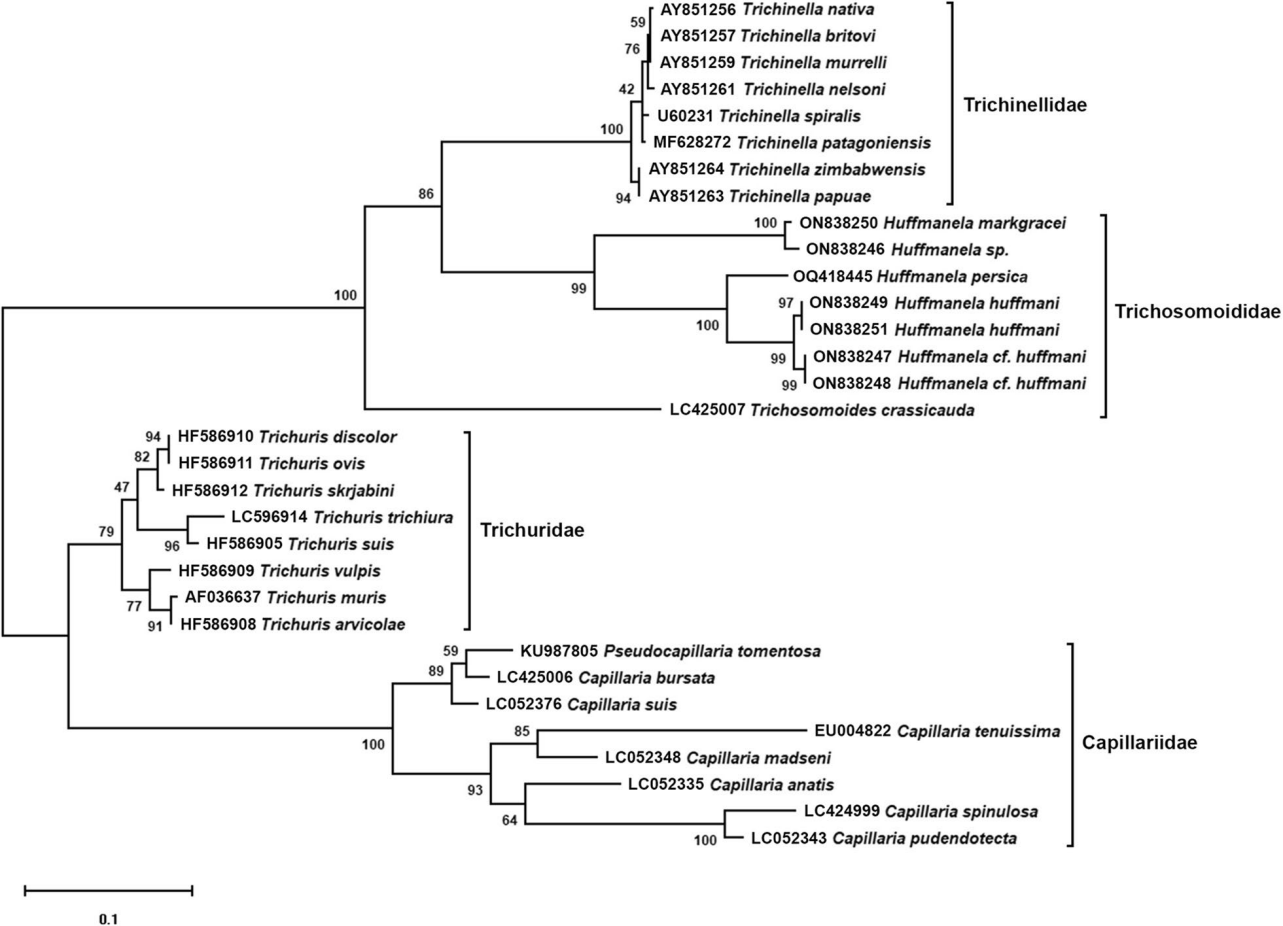
Maximum likelihood (ML) phylogram reconstructed using the 18S rDNA dataset of the new species *Huffmanela persica* sp. nov. and other related species within the families Trichosomoididae, Trichinellidae, Trichuridae and Capillariidae. ML analysis was performed using the substitution model K2 + G with 1000 bootstrap replications

Sequencing of purified PCR products of 18S, 28S and ITS regions from *H. persica* sp. nov. resulted in DNA fragments with different lengths (855 bp, 1264 bp and 1127 bp, respectively). The BLAST analysis revealed that 18S rDNA gene sequence of *H. persica* sp. nov. shared 93–96% identity (ID) and 83–96% query coverage (QC) with *Huffmanela* species collected from freshwater bony fish including *H.* cf. *huffmani* of Bullard et al., 2022 (ON838248, 93.12% ID and 96% QC), *H. huffmani* (ON838249, 94.62% ID and 88% QC), *H. huffmani* (ON838251, 96.08% ID and 83% QC) and *H.* cf. *huffmani* (ON838247, 95.80% ID and 83% QC) and 86–89% ID and 81–95% QC with those isolated from marine cartilaginous fish including *Huffmanela* sp. (ON838247, 86.28% ID and 95% QC) and *Huffmanela markgracei* Ruiz and Bullard, 2013 (ON838250, 89.38% ID and 81% QC). According to the ML tree generated from 18S rDNA sequences (Fig. 6), *H. persica* was not grouped with previously reported species of *Huffmanela*. However, a sister relationship was recovered between the new species of marine origin with freshwater species of *Huffmanela* (*H. huffmani* and *H.* cf. *huffmani*), all of which are known to infect teleost fish. A sister group relationship was also found between *Huffmanela* species infecting teleost fish (*H. huffmani*, *H.* cf. *huffmani* and *H. persica*) with those parasitizing elasmobranch fish (*H. markgracei* and *Huffmanela* sp.). Phylogenetic analysis based on 18S rDNA dataset strongly corroborated monophyly of the subfamily Huffmanelinae (Fig. 6).

## Discussion

The description and examination of developing eggs of *Huffmanela* provide valuable information regarding the taxonomy and biodiversity of the genus [38, 39]. However, differential comparison of various species of the genus *Huffmanela* based on the eggs has been suggested to be principally performed according to parasitological analyses of fully developed eggs with consideration of host-parasite interactions [15, 39]. Accordingly, a combination of diagnostic characteristics has been previously applied for differentiation of *Huffmanela* species. These include morphometric analyses (egg measurements), general morphology (color, shape, structure, surface ornamentation), host species, preferred site of infection in the host’s body (specific tissues infected by the parasite) and geographical locality. *Huffmanela persica* sp. nov. can be principally distinguished from the other accepted and unnamed species of *Huffmanela* by having a range of features including different size of egg proper, distinct ornamentation of UL and rarely observed eggshell adornment. The dimensions (considering both length and width) of advanced eggs (54–68 × 31–43 µm) of the new species closely or partly resemble those of *H. huffmani* (54–60 × 30–33 µm) [1], *H.* cf. *huffmani* (54–66 × 27–33 µm) [5], *H. markgracei plectropomi* Justine, 2011 (64–76 × 29–35 µm) [40], *H. hamo* Justine and Iwaki, 2014 (65–78 × 33–38 µm) [12], *Huffmanela moraveci* Carballo and Navone, 2007 (50–57 × 23–31 µm) [41], *H. longa* Justine, 2007 (58–72 × 22–34 µm) [2], *H. balista* Justine, 2007 (63–78 × 32–41 µm) [2], *H. mexicana* Moravec and Fajer-Avila, 2000 (63–66 × 30–33 µm) [22], *H. selachii* Al-Sabi et al., 2022 (63–90 × 30–56 µm) [42] and *Huffmanela* sp. of Attia et al., 2021b (62–75 × 30–36 µm) [43]. On the other hand, as observed in *H. persica* sp. nov. in the form of protuberance and mammiform mounds, distinctively shaped ornamentations associated with eggshell or UL have been previously described for *Huffmanela schouteni* Moravec and Campbell, 1991 (UL with protuberances) [44], *H. banningi* Van Banning, 1980 (UL apparently spinose) [20], *H. huffmani* [1], *H. japonica* Moravec et al., 1998 (eggshell with protuberances) [18], *H. canadensis* Moravec et al., 2005 (eggshell with transverse ridges) [45], *H. lata* Justine, 2005 (eggshell apparently spinose) [39], *H. balista* (eggshell with longitudinal ridges) [2], *H. moraveci* (eggshell spinose) [41], *H. markgracei* (shell with transverse ridges) [46], *H. oleumimica* Ruiz et al., 2013 (eggshell spinous) [15], *Huffmanela* sp. (eggshell with protuberance) [43], *H.* cf. *huffmani* (UL with spines) [5] and *H. psittacus* Carvalho et al., 2022 (shell with longitudinal, oblique and transverse ridges) [6]. Of the abovementioned species, *H. persica* sp. nov. can be easily differentiated from *H. schouteni*, *H. banningi*, *H. oleumimica*, *H. markgracei*, *H. lata* and *H. psittacus* by having dissimilar size of eggs (Table 3). Likewise, the eggs of *H. persica* sp. nov. are wider compared to those of *H. japonica* and *H. moraveci*. The eggs of new species are remarkably different from those of *Huffmanela plectropomi*, *H. hamo*, *H. longa*, *H. mexicana*, *H. japonica*, *H. canadensis*, *H. moraveci*, *H. ballista*, *H. selachii* and *Huffmanela* sp. [43] by possessing unique surface structures (eggshell with protuberances and UL with mammiform mounds adorned with a tendril-like vermiform appendage in *H. persica* sp. nov. versus smooth eggshell in *H. hamo*, *H. longa*, *H. mexicana* and *H. selachii*; eggshell with transverse ridges in *H. canadensis*; eggshell with longitudinal ridges in *H. balista*; absence of UL in *H. plectropomi*, *H. hamo*, *H. canadensis* and *H. selachii*; smooth UL in *H. japonica*, *H. moraveci*, *H. balista* and *Huffmanela* sp. of Attia et al., 2021b). Egg measurements of *H. huffmani* and *H.* cf. *huffmani* [5] closely overlap those of the new species. Nevertheless, these species of freshwater origin can be separated from the marine species *H. persica* sp. nov. depending upon the characteristics such as eggshell without protuberances (versus eggshell with protuberances) and distinct egg UL with papilla-like spines (versus UL with mammiform mounds). *Huffmanela huffmani* and *H.* cf. *huffmani* were respectively described from freshwater fish within the families Centrarchidae and Poeciliidae in the USA [3, 5].

**Table 3:** Comprehensive list of nominal and unidentified species of *Huffmanela* Moravec, 1987, reported in previous studies in chronological order

	Egg size^a,*^ L × W	Plug size^b,*^ L × W	Shell thickness^*^	Shell (wall) surface	Uterine layer (UL)	Larval size^c,*^ L × W	Adult (male and female) size^*^ L × W	Host and family	Infected tissue(s)	Ecosystem	Locality	References
*Huffmanela carcharhini*	75–105 × 42–63	— × 8–13	5–10	Smooth (possibly with minute spines)	NF or visible at pole regions	188–273 × 8–13	ND	*Carcharhinus melanopterus*; *Carcharhinus plumbeus* (Carcharhinidae)	Skin, gill arches	Marine	Atlantic Ocean (USA); Pacific Ocean (USA)	[1, 10, 69]
*Huffmanela schouteni*	69–75 × 27–33	9–12 × 6–9	3–5	Smooth	Thin UL with protuberances	210 × 4–6	ND	*Hirundichthys affinis*; *Cheilopogon cyanopterus*; *Cheilopogon heterurus* (Exocoetidae)	Abdominal cavity, intestinal serosa, liver, swim bladder	Marine	Curaçao (the Netherlands); Ligurian Sea (Italy)	[19, 44, 70]
*Huffmanela banningi*	99–108 × 42–50	— × 9	3	Smooth	Apparently spinose	350–400 × 15	ND	*Cynoglossus browni* (Cynoglossidae)	Muscle	Marine	Atlantic Ocean (Senegal and Congo)	[1, 20]
*Huffmanela* sp.	113–116 × 60	ND	ND	Smooth	Delicate transparent UL	ND	ND	*Genypterus blacodes* (most likely) (Ophidiidae)	Muscle	Marine	Pacific Ocean (New Zealand)	[38, 71]
*Huffmanela huffmani*	54–60 × 30–33	9 × 6–7	4.5–6	Smooth	UL with minute spines	— × 6	4690–5140 × 24–27 (M); 4900–7510 × 24–30 (F)	*Lepomis* spp.; *Ambloplites rupestris*; *Micropterus salmoides* (Centrarchidae)	Swim bladder	Freshwater	San Marcos River, Texas (USA)	[1, 3, 9, 14, 72, 73]
*Huffmanela* sp.	67–79 × 34–43	7–8 × 16–19	2–3	Smooth	Thin transparent UL	220–240 × 8	ND	Non-fish host (false parasite of human)	Found in human feces	ND	Palma de Mallorca (Spain)	[17]
*Huffmanela japonica*	58–69 × 26–30	6–9 × 6–9	4–5	Transparent protuberances	Transparent UL with smooth surface	— × 9	ND	*Upeneus bensasi* (Mullidae)	Muscle	Marine	Inland Sea of Japan, off Shikoku Island (Japan)	[18]
*Huffmanela shikokuensis*	78–90 × 36–45	8–12 × 15–18	3	Smooth	UL poorly developed with smooth surface	— × 9	— × 21–33 (by histological section)	*Stephanolepis cirrhifer* (Monacanthidae)	Muscle	Marine	Inland Sea of Japan, off Shikoku Island (Japan)	[18]
*Huffmanela paronai*	48–51 × 21–24	6 × 6–7	3	Smooth	Transparent UL with smooth surface	ND	ND	*Xiphias gladius* (Xiphiidae)	Skin	Marine	Ligurian Sea (Italy)	[11]
*Huffmanela mexicana*	63–66 × 30–33	6 × 6	4–5	Smooth	UL poorly developed with smooth surface	— × 6	ND	*Sphoeroides annulatus* (Tetraodontidae)	Swim bladder	Marine	Mazatlán, Sinaloa State, Mexico	[22]
*Huffmanela canadensis*	48–63 × 21–27	6–9 × 6	3	Transverse ridges	NF	ND	3441–3862 × 45–63 (M); 7711–8160 × 90–105 (F)	*Sebastes* spp. (Scorpaenidae)	Skin	Marine	Pacific Ocean off the coast of Vancouver Island (Canada)	[45, 59]
*Huffmanela branchialis*	45–52 × 23–30	ND	2–3	Smooth	Spindle-shaped UL with smooth surface	150 × 6–7	ND	*Nemipterus furcosus* (Nemipteridae)	Gill	Marine	Pacific Ocean, off New Caledonia (France)	[74]
*Huffmanela filamentosa*	48–53 × 25–30	ND	3	Smooth (long thin filaments at egg extremity)	NF	150 × 7	ND	*Gymnocranius grandoculis* (Lethrinidae)	Gill	Marine	Pacific Ocean, off New Caledonia (France)	[74]
*Huffmanela ossicola*	73–88 × 35–40	ND	8–10	Smooth	Thin UL encompassing numerous thin filaments	250 × —	ND	*Bodianus loxozonus* (Labridae)	Bone	Marine	Pacific Ocean, off New Caledonia (France)	[74]
*Huffmanela* sp.	39–47 × 22–27	ND	3	ND	NF	ND	ND	*Pentapodus aureofasciatus* (Nemipteridae)	Mucosa of the mouth; gill	Marine	Pacific Ocean, off New Caledonia (France)	[74]
*Huffmanela lata*	77–88 × 52–63	ND	6–8	Irregular, apparently spinose	NF	220–250 × 8	ND	*Carcharhinus amblyrhynchos* (Carcharhinidae)	Skin	Marine	Pacific Ocean, off New Caledonia (France)	[39]
*Huffmanela* sp.	70–86 × 34–47	0.6–4.1 × 3.8–12.3	3.3–6.6	Smooth with thickened chitinous layer at both ends	Thin transparent UL with smooth surface	— × 6.2–9.4	ND	*Carcharhinus plumbeus* (Carcharhinidae)	Skin	Marine	Atlantic Ocean, North Carolina (USA)	[75]
*Huffmanela balista*	63–78 × 32–41	ND	5–6	Longitudinal ridges	Thin UL with smooth surface	245–295 × 6.5	9870 × 30 (M)	*Abalistes stellatus* (Balistidae)	Swim bladder	Marine	Pacific Ocean, off New Caledonia (France)	[2]
*Huffmanela longa*	58–72 × 22–34	ND	2–4	Apparently smooth (long thin filaments at egg extremity)	NF	140–168 × 4–5	— × 32 (F)	*Gymnocranius grandoculis* (Lethrinidae)	Mesentery, mucosa of abdominal cavity, swim bladder	Marine	Pacific Ocean, off New Caledonia (France)	[2]
*Huffmanela moraveci*	50–57 × 23–31	6–8 × 8–10	3–5	Spinous	Smooth	108 × —	3800–6400 × 60–90 (M); 5000–16,300 × 58–120 (F)	*Odontesthes* spp. (Atherinopsidae)	Skin, gill	Marine	Nuevo and San José gulfs, Atlantic Ocean (Argentina)	[41]
*Huffmanela* sp.	73.4–94.3 × 39.6–59.1	— × 14–22	ND	Smooth	NF	— × 8.6–12.9	ND	*Trisopterus luscus* (Gadidae)	Muscle	Marine	Atlantic Ocean off the Portuguese coast (Portugal)	[60]
*Huffmanela* sp.	ND	ND	ND	ND	ND	ND	6460 × 48 (F)	*Bodianus perditio* (Labridae)	Digestive tract	Marine	Pacific Ocean, off New Caledonia (France)	[76]
*Huffmanela plectropomi*	64–76 × 29–35	— × 6	3–4	Surrounded by filamentous layer	NF	ND	ND	*Plectropomus leopardus* (Serranidae)	Mesentery near swim bladder wall	Marine	Pacific Ocean, off New Caledonia (France)	[40]
*Huffmanela markgracei*	99–109 × 39–49	13–22 × 8–12	3–5	Transverse ridges	Smooth	255–335 × 8–10	ND	*Rhizoprionodon terraenovae* (Carcharhinidae)	Skin, tongue, gill arches, buccal cavity	Marine	Gulf of Mexico off Texas, (USA)	[46]
*Huffmanela oleumimica*	46–54 × 23–33	6–8 × 6–9	2–3	Spinous	Smooth	160–201 × 7–8	ND	*Lutjanus campechanus* (Lutjanidae)	Skin	Marine	Gulf of Mexico off Texas, (USA)	[15]
*Huffmanela hamo*	65–78 × 33–38	ND	5–6	Smooth	NF	ND	ND	*Muraenesox cinereus* (Muraenesocidae)	Somatic muscle	Marine	Off Japan (Japan)	[12]
*Huffmanela* sp.	85 × 45	ND	ND	ND	ND	ND	ND	*Sphyrna tiburo* (Sphyrnidae)	Skin	Marine	Off the Florida coast, USA	[61]
*Huffmanela* sp.	74.77–86.2 × 36.86–42.27	4.82–6.87 × 14.27–19.82	ND	Smooth	Thin transparent UL	— × 7.4–10.04	ND	*Microchirus azevia* (Soleidae)	Muscle, skin, connective tissue	Marine	Atlantic Ocean (Portugal)	[62]
*Huffmanela lusitana*	67.6–77.2 × 36.1–47.1	0.7–8.6 × —	1.7–3	Smooth	Smooth	370 × —	ND	*Trisopterus luscus* (Gadidae)	Muscle	Marine	Atlantic Ocean off the Portuguese coast (Portugal)	[16]
*Huffmanela.* sp.	ND	ND	ND	ND	ND	ND	6801 × 24 (M); 10,201 × 80 (F)	*Acanthopagrus bifasciatus* (Sparidae)	Mesentery	Marine	Persian Gulf off Iraq (Iraq)	[51]
*Huffmanela* sp.	92- 110 × 34–49	— × 4.5–11.5	1.5- 2.2	Smooth	Smooth	ND	ND	*Carcharhinus dussumieri* (Carcharhinidae)	Skin	Marine	Persian Gulf off Jubail (Saudi Arabia)	[50]
*Huffmanela* sp.	62.5–75.3 × 30–36.5	— × 6.3–9.2	3–5.4	Shell surface with protuberance	Smooth	— × 4.5–6	ND	*Epinephelus coioides* (Serranidae)	Muscle	Marine	Persian Gulf off Jubail (Saudi Arabia)	[43]
*Huffmanela* sp.	ND	ND	ND	Smooth	NF	ND	ND	*Cheilinus lunulatus* (Labridae)	Swim bladder	Marine	Red Sea off Hurghada (Egypt)	[63]
*Huffmanela* cf.* huffmani*	54–66 × 27–33	6–9 × 6–9	3–6	Smooth	Transparent UL with minute papillae (spines)	ND	3790–12,920 × 21–42 (M); 5810–14,990 × 21–36 (F)	*Xiphophorus variatus; Gambusia holbrooki* (Poeciliidae)	Swim bladder, gonad (testis), peritoneum	Freshwater	West central Florida (USA)	[5]
*Huffmanela psittacus*	46.7–51.7 × 21.7–25	2.30–6.70 × 8.30–10	ND	Shell surface with longitudinal, oblique and transverse ridges	ND	ND	3400–5600 × 18–28 (M); 7500–11,900 × 30–48 (F)	*Colomesus psittacus* (Tetraodontidae)	Gills, gill arch, gill mucosa	Freshwater (euryhaline fish)	Marajó Island, Pará State (Brazil)	[6]
*Huffmanela selachii*	62.69–89.9 × 29.8–56.1	— × 4.5–16	ND	Smooth	NF	ND	ND	*Sphyrna mokarran* (Sphyrnidae); *Carcharhinus melanopterus* (Carcharhinidae)	Skin	Marine	Persian Gulf off Jubail (Saudi Arabia)	[42]
*Huffmanela persica* sp. nov	54.37–67.86 × 30.96–43.39	— × 8.43–12	3.51–6.11	Shell surface with irregular protuberances	UL with mammiform mounds with tendril-like vermiform appendage	196–231 × 6–9	ND	*Muraenesox cinereus* (Muraenesocidae)	Gonad (ovary), tunica serosa of stomach	Marine	Persian Gulf off the coast of Zir Ahak, Bushehr (Iran)	Present study

*L* length, *W* width, *ND* not described, *NF* not found (by the authors in the study performed)

^a^Size of eggs is according to the size of eggshell proper in fully developed (advanced) eggs (without envelope and protruding part of polar plugs)

^b^Polar plug length (height) without protruding part

^c^Maximum body width reported

^*^All measurements associated with different forms of the nematode (eggs, larvae and adults) are provided in micrometer (µm) as minimum and maximum measurements reported (if provided)

*Muraenesox cinereus* is an oceanodromous fish species widely distributed in the Western Indo-Pacific realm including the Persian Gulf [47–49]. Previous reports are available on the infection of marine teleost and elasmobranch fish collected from the Persian Gulf region with nominal (*H. selachii*) [42] and unnamed species of *Huffmanela* (*Huffmanela* sp. of Attia et al., 2021a, *Huffmanela* sp. of Attia et al., 2021b, and *Huffmanela* sp. of Al-Hasson et al., 2019) [43, 50, 51]. However, *H. persica* sp. nov. is distinguishable from these species on the basis of egg size, egg overlays, infected tissues and host fish (Table 3). Justine and Iwaki (2014) described the eggs of *H. hamo* from the somatic musculature of the anguilliform fish *M. cinereus* (off Japan), which as previously stated is morphologically different from the new species [12]. In this study, however, eggs of *H. persica* sp. nov. were detected in the ovary and serosa of stomach of the same species, suggesting that tissues parasitized by different populations of *Huffmanela* are species specific in this genus [11, 45]. The biology of larval and adult forms of the daggertooth pike conger is largely unknown. No food material has ever been detected in the intestine of any anguillid leptocephali inhabiting natural waters. As concluded by Hulet (1978), the gut is poorly differentiated in anguilliform leptocephali, and protoplasmic juices from organisms punctured by the projecting teeth could be absorbed through the epithelium of the esophagus and stomach in larval stages [52]. In a previous study, no captive-reared larvae of pike conger were found to strike at foods such as Chlorella, rotifers, Protozoa, fertilized eggs of sea urchin and phytoplankton [53]. However, pike conger of larger size is an active predator feeding on the bathypelagic-pelagic, demersal and small bottom living organisms including fishes, crustaceans and cephalopods [54–56]. The stomach content of pike congers collected from Porto-Novo, West Africa, was found to be predominantly composed of mackerel, Clupeids, *Caranx* species, squids and flying fish [57]. Another study also revealed that *Engraulis japonicus* (Engraulidae) was the main preferred prey of pike congers collected from the coastal waters off Goseong, South Korea [58]. Unfortunately, there is a dearth of studies on habitat use, feeding habits and seasonal dietary preferences of pike conger and its preferred prey in the Persian Gulf, and therefore further studies are required to determine whether intermediate or paratenic hosts are involved in the life cycle of *H. persica* sp. nov.

Masses of eggs of *H. persica* sp. nov. were observed in the ovary and the tunica serosa of the stomach in infected fish. Similarly, tissues with a serous membrane were infected with eggs of *H. schouteni* (intestinal serosa) [44], *H. longa* (mesentery) [2], *H. plectropomi* (mesentery near swim bladder) [40], *H.* cf. *huffmani* (visceral peritoneum) [5] and *Huffmanela* sp. (mesentery) [51]. Infection with eggs of *H.* cf. *huffmani* was also detected in the gonad (testes) of platyfish (*Xiphophorus variatus*) [5]. In this study, eggs of *H. persica* were found to be surrounded by epithelioid cells and eosinophilic granulocytes, implicating the inflammatory response resulted from infection with the parasite. Immunopathological responses induced by intercellular and intracellular infection with eggs or worms of different species of *Huffmanela* have been previously reported in various fish hosts, reflecting the physical and chemical damage caused by the parasite to the infected tissues. These include granulomatous inflammation (characterized mainly by infiltration of mononuclear phagocytes), inter-cellular edema (spongiosis), cellular adaptive response (hypertrophic, atrophic and hyperplastic changes), degenerative and necrotic changes and degenerative and dystrophic calcification [5, 6, 10, 16, 43, 59–63].

The superfamily Trichinelloidea currently comprises five families including Capillariidae, Trichinellidae, Trichuridae, Cystoopsidae and Trichosomoididae [33, 38]. According to Moravec (2001), the family Trichosomoididae includes three subfamilies, Anatrichosomatinae Smith and Chitwood, 1954, Huffmanelinae Moravec, 1987, and Trichosomoidinae Hall, 1916 [38]. Anatrichosomatinae and Huffmanelinae contain only one genus, namely *Anatrichosoma* (Smith and Chitwood, 1954) and *Huffmanela*, whereas Trichosomoidinae represents two genera, *Trichosomoides* (Railliet, 1895) and *Trichuroides* (Ricci, 1949). Hodda (2022) recently listed the genus *Paratrichosoma* Ashford and Muller, 1978, in the family Trichosomoididae [33]; however, the genus was previously suggested to be the synonymy of *Capillaria* and placed in the family Capillariidae [24, 38, 64, 65]. In this study, phylogenetic analysis based on the 18S rDNA sequences recovered a sister relationship between *Huffmanela* and *Trichinella* (Railliet, 1895). Sister group relationship between these two genera was also supported by Bayesian phylogenetic analysis in a previous study [5]. Phylogenetic relationships among species of the genus *Huffmanela* revealed two distinct clades with maximum support. The first clade, Chondrichthyes (elasmobranch)-associated clade, includes *Huffmanela* species (*H. markgracei* and *Huffmanela* sp.) collected from marine elasmobranch fish, whereas the second clade, Osteichthyes (teleost)-associated clade, represents *Huffmanela* species infecting marine (*H. persica*) and freshwater (*H. huffmani* and *H.* cf. *huffmani*) teleost fish. However, further nucleotide sequences representing the nuclear and mitochondrial rRNA genes from marine species of *Huffmanela* infecting teleost fish are required to elucidate whether freshwater species of *Huffmanela* are derived from marine ancestors [66]. If freshwater species of *Huffmanela* presented a monophyletic group that is nested within a group of marine taxa, it may support the hypothesis that the genus *Huffmanela* might have a marine origin [5, 67, 68].

## Conclusions

To the best of our knowledge, the present study provides the molecular sequences from the 28S and ITS regions of nuclear rDNA in a species belonging to the genus *Huffmanela* for the first time. The phylogenetic position of a marine species of *Huffmanela* was demonstrated for the first time based on analysis of the 18S rDNA sequence data obtained from the new species and those previously described from freshwater species of *Huffmanela*.

## Data Availability

The datasets used and/or analyzed during the current study are available from the corresponding author upon reasonable request. Nucleotide sequences of the 18S rDNA (OQ418445), 28S rDNA (OQ428648) and ITS rDNA (OQ428649) of the new species have been deposited in GenBank.

## Abbreviations

18S: Small subunit ribosomal DNA
28S: Large subunit ribosomal DNA
ID: Identity
ITS: Internal transcribed spacer
HMDS: Hexamethyldisilazane
LSID: Life Science Identifier
ML: Maximum likelihood
PCR: Polymerase chain reaction
QC: Query coverage
rDNA: Ribosomal DNA
UL: Uterine layer
